# OneNet—One network to rule them all: Consensus network inference from microbiome data

**DOI:** 10.1371/journal.pcbi.1012627

**Published:** 2024-12-06

**Authors:** Camille Champion, Raphaëlle Momal, Emmanuelle Le Chatelier, Mathilde Sola, Mahendra Mariadassou, Magali Berland

**Affiliations:** 1 Université Paris-Saclay, INRAE, MGP, Jouy-en-Josas, France; 2 Université Paris-Saclay, INRAE, MaIAGE, Jouy-en-Josas, France; Genome Institute of Singapore, SINGAPORE

## Abstract

Modeling microbial interactions as sparse and reproducible networks is a major challenge in microbial ecology. Direct interactions between the microbial species of a biome can help to understand the mechanisms through which microbial communities influence the system. Most state-of-the art methods reconstruct networks from abundance data using Gaussian Graphical Models, for which several statistically grounded and computationnally efficient inference approaches are available. However, the multiplicity of existing methods, when applied to the same dataset, generates very different networks. In this article, we present OneNet, a consensus network inference method that combines seven methods based on stability selection. This resampling procedure is used to tune a regularization parameter by computing how often edges are selected in the networks. We modified the stability selection framework to use edge selection frequencies directly and combine them in the inferred network to ensure that only reproducible edges are included in the consensus. We demonstrated on synthetic data that our method generally led to slightly sparser networks while achieving much higher precision than any single method. We further applied the method to gut microbiome data from liver-cirrothic patients and demonstrated that the resulting network exhibited a microbial guild that was meaningful in terms of human health.

## Author summary

Exploring how microbes interact within our intestines is a fascinating challenge in microbial ecology. Understanding these interactions helps us uncover the ways microbial communities influence both microbiota composition and the host system. New methods that reconstruct microbial network are published every year, but when different methods are used on the same data, they produce varying outcomes. There is currently no consensus on the best method to use. Our study introduces “OneNet” a new approach that combines seven methods to create a unified network. Our method showed on simulated data that it produced simpler networks while improving accuracy compared to the existing methods. On real gut microbiome data from liver cirrhosis patients, we identified in the network a cirrhotic cluster, composed of bacteria associated with degraded host clinical status. Our research contributes to unraveling the microbial interactions, potentially leading to better understanding of the role of the gut microbiota on health.

## Introduction

The human gut microbiota is a complex ecosystem consisting of trillions of microorganisms, mainly viruses, bacteria, archeae and microbial eucaryotes, that play critical roles in host physiology including digestion, immune function and metabolism [1, 2]. Recent advances in sequencing technologies have enabled the characterization of gut microbiota composition and function at a fine scale, providing opportunities to understand the microbial communities that reside within the human gastrointestinal tract. However, despite these technological advancements, understanding the interactions within the bacteria of the gut microbiota remains a major challenge. These interactions are complex as microorganisms can interact with each other in a multitude of ways: through mutualism, parasitism, commensalism and competition to only cite a few [3, 4].

To address this challenge, network-based approaches have been developed to infer microbial interactions and construct microbial interaction networks. The resulting networks can reveal potential interactions between microbial taxa and support the identification of microbial guilds. Those guilds are defined as groups of microorganisms that co-occur and may interact with each other. Identifying microbial guilds is crucial for understanding the ecological dynamics of the gut microbiota and can provide insights into the role of the microbiota in health and disease [5, 6].

Formally, microbial interaction networks consist of nodes, which correspond to microbial species, and edges, which correspond to interactions between those species. Positive and negative interactions are rarely observed directly. They are instead often reconstructed from abundance data, using either longitudinal data (see the generalized Lotka-Volterra model in [7]) or co-occurrence data. We focus here on the latter suite of methods.

The simplest way to identify microbial interactions is to perform a correlation analysis. However, correlation-based methods model total dependencies and are therefore prone to confusion by environmental factors (*e.g.* shared habitat preferences or susceptibility to the same abiotic factors) and do not lend themselves to a clear separation between indirect and direct effects [8]. By contrast, conditional dependency-based method eliminate indirect correlations from direct interactions and lead to sparser and easier to interpret networks, at the cost of increased computational burden and more sophisticated models. The problem of network inference is complicated by the adverse characteristics of microbial abundance data, which are sparse, heterogeneous, heteroscedastic and show extreme variability. These data are thus tricky to model and lead to poorly reproducible and/or sparse networks, with many missing edges [9].

**Fig 1:**
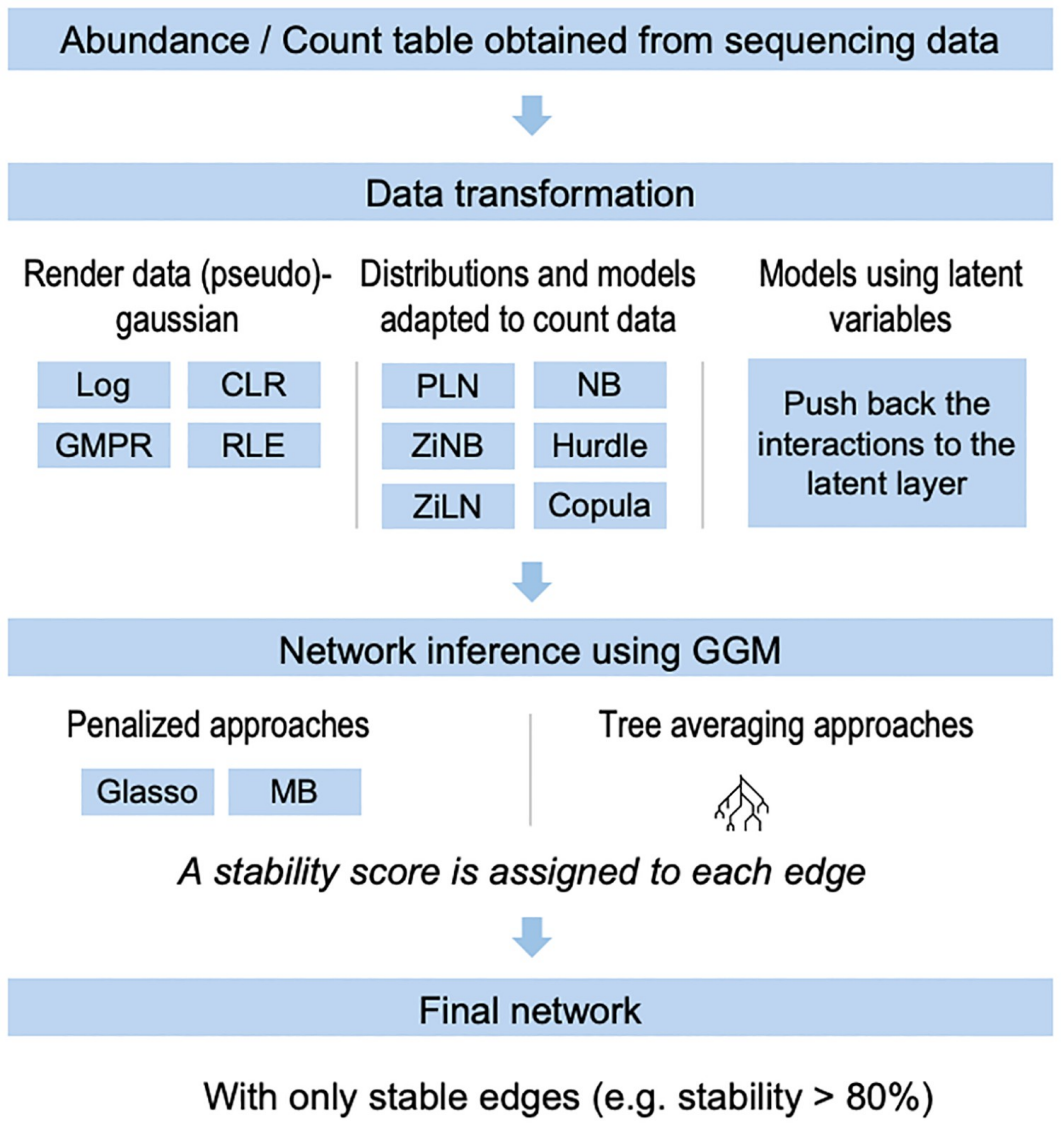
The classical network inference pipeline.

The most common framework for the estimation of the conditional dependencies is Gaussian Graphical Models (GGM) [10], which describe the conditional dependency structure of multivariate Gaussian distributions. As microbiome abundance data don’t directly fit within the gaussian framework, three main workarounds are commonly used: data transformation, models based on alternative distributions and models based on latent variables; the whole strategy is also illustrated on Fig 1.

The complexity of reconstructing networks from co-occurrence data has spawned a rich literature with many methods relying on the solutions exposed above, including (i) approaches based on correlation as SparCC [8], CoNet [4], (ii) approaches based on probabilistic graphical models as SpiecEasi [11], gCoda [12], SPRING [13], PLNnetwork [14], ZiLN [15], COZINE [16], Magma [17], EMtree [18] and (iii) approaches based on the inference of the latent correlation structure as CCLasso [19], SparCC [8]. The most recent methods include: mixPLN and ms-mixPLN [20, 21] which consider the problem of inferring multiple microbial networks (one per host condition) from a given sample-taxa abundance matrix when microbial associations are impacted by host factors. The HARMONIES approach [22] addresses some critical aspects of abundance data (compositionality due to fixed sampling depth, over-dispersion and zero-inflation of the abundances) while maintaining computational scalability and sparsity of the interaction network, in contrast to mixPLN and ms-mixPLN. Finally, NetCoMi [9], provides a one-step platform for inference and comparison of microbial networks, by implementing many existing methods for abundance data preprocessing, network inference and edge selection in a single package.

All these methods have been designed to infer networks based on different mathematical hypotheses and thus have different strengths and weaknesses when modeling microbiome data. Each microbial network inference algorithm usually returns distinct edges to connect the taxa together, as many facets of the same reality. Methods to combine different microbial networks have been proposed before but they rely on spectral decomposition and do not work at the edge level [23]. In this article, we present OneNet, an ensemble method that generates robust and reliable consensus network that will facilitate the identification of microbial guilds and generation of new hypotheses.

## Methods

### Overview of OneNet

**Fig 2:**
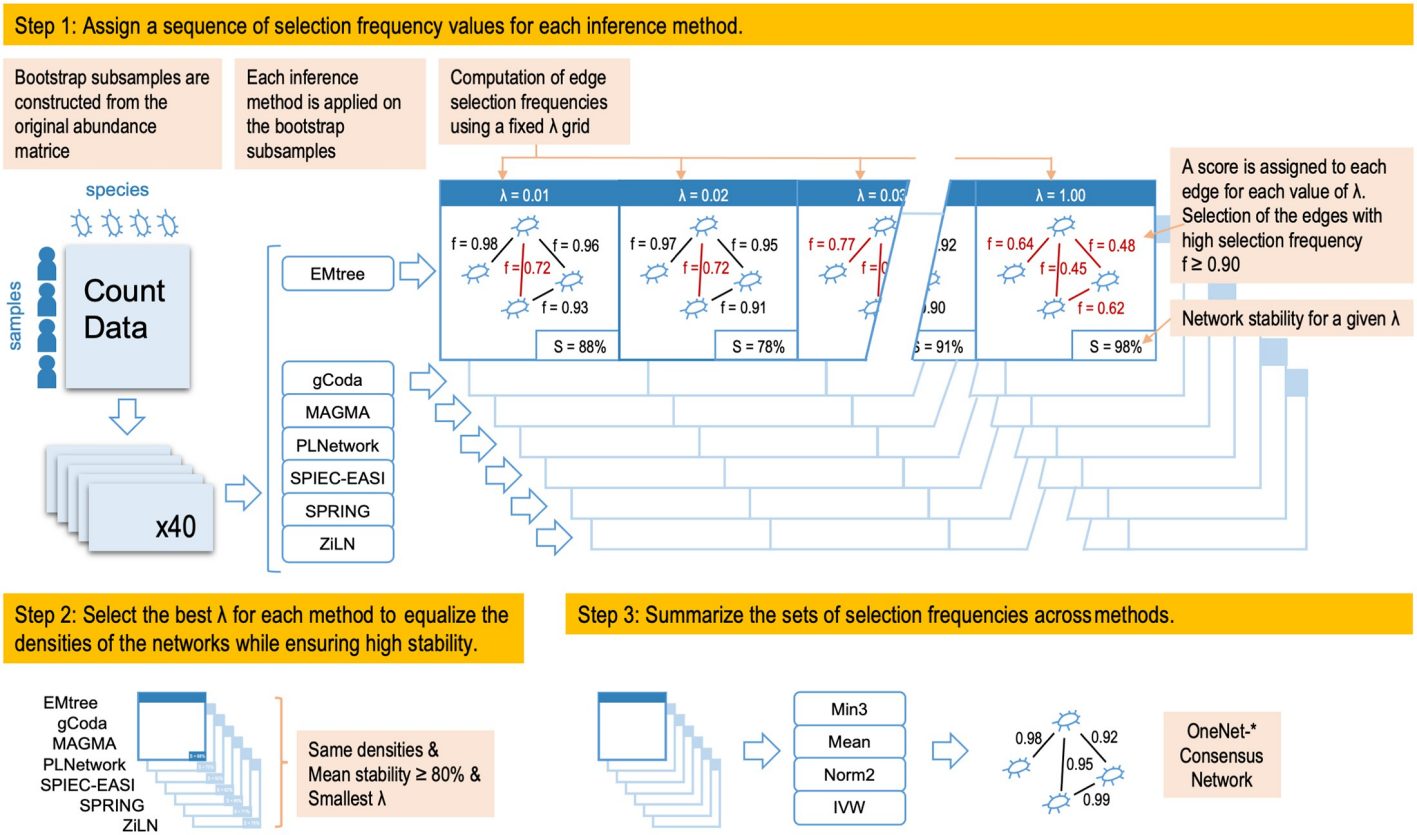
High level summary of the OneNet pipeline. (i) bootstrap subsamples are constructed from the original abundance matrix, (ii) each inference method is applied on the bootstrap subsamples to compute edge selection frequencies using a fixed λ grid, (iii) a different λ is selected for each method to achieve the same density in all methods, (iv) edge selection frequencies are summarized and (v) thresholded to compute the consensus graph.

We developed OneNet, a three-step procedure for robust consensus network reconstruction based on abundance microbial data at any taxonomic rank, illustrated on Fig 2.

We included seven inference methods in OneNet, all of which rely on Gaussian Graphical Models (GGM) to estimate conditional dependencies networks: Magma, SpiecEasi, gCoda, PLNnetwork, EMtree, SPRING and ZiLN. We excluded mixPLN and ms-mixPLN as they do not reconstruct a single network but rather a collection of networks and NetCoMi as it collects already existing methods rather than introducing a new one. We left out HARMONIES from the comparison as its implementation doesn’t allow the user to specify the regularization grid, a crucial step in the ensemble method, and achieved worse performance than included methods in preliminary tests. We also excluded COZINE from OneNet because its implementation doesn’t rely on resampling and prevents it from being integrated, but we nonetheless included it in the benchmark as it compared favorably to others methods in preliminary tests. Table 1 summarizes the inference strategies adopted by each method and the potential integration of covariables in the model.

**Table 1:** Characteristics of the network inference methods.

Method	Normalization	Distribution	Inference approach	Covariates	Reference
SpiecEasi	CLR	Multivariate gaussian	MB	No	[11]
gCoda	CLR	Multivariate gaussian	glasso	No	[12]
SPRING	CLR	Copulas	MB	No	[13]
Magma	GMPR + RLE	Copulas + ZINB	MB	Yes	[17]
PLNnetwork	GMPR + RLE	PLN + Latent variables	glasso	Yes	[14]
EMtree	GMPR + RLE	Latent variables	Tree averaging	Yes	[18]
ZiLN	CLR	Latent variables	MB	No	[15]
*COZINE*	CLR	Hurdle gaussian	MB	Yes	[16]

Inference methods, abundances normalization (Centred Log Ratio (CLR), Geometric Mean of Pairwise Ratios (GMPR), Relative Log Expression (RLE)), distribution transformations, inference approaches (Meinshausen-Bühlmann (MB), glasso, tree averaging), covariates integration and references. OneNet is based on all methods but *COZINE*.

### Step 1: Assign a sequence of selection frequency values to each edge for each inference method

Each inference method assigns a score to the edges: either a probability (for the tree averaging method) or the maximal penalty level λ above which the edge is selected in the network. An optimal penalty λ^*^ on these scores is then needed for an edge to be selected in the final network. Several approaches exist but the concept of stability selection [24] is the most widely used and the one considered in this work as it yields a compromise between precision and recall, while fostering reproducibility. The associated method, called Stability Approach to Regularization Selection (StARS) uses a resampling strategy to select the value of λ^*^ leading to the most stable graph. We describe briefly in the following the StARS algorithm introduced by [24] before presenting the modification of StARS we propose in this work.

#### StARS algorithm (from [24])

The full abundance matrix, *X*, where samples and taxa are indexed in *n* rows and *p* columns is subsampled *B* times by selecting a subset of rows to create *B* subsamples *X*^(1)^, …, *X*^(*B*)^, each of size *n*^′^ = 0.8*n* if *n* ≤ 144 and $n^{\prime }=10\sqrt{n}$ otherwise, following the recommendations of [24]. The network inference is conducted on each subsample *X*^(*b*)^ for each value of λ in a grid (λ_1_, …, λ_*K*_) to obtain a graph *G*^*b*, *k*^, with *k* ∈ {1, …, *K*} and *b* ∈ {1, …, *B*}. The selection frequency of edge *e* for parameter λ_*k*_, is computed as its selection frequency across the subgraphs:

$$\begin{array}{c} f_{e}^{k}=\frac{1}{B}\sum_{b=1}^{B}1_{\left\{e\in G^{b,k}\right\}}. \end{array}$$

where 1_{*a* ∈ *A*}_ is the indicator function for *a* to belong to set *A*. The selection frequency over resamples gives an idea of edge reproducibility: frequency and robustness of the edges are clearly related. StARS aggregate those frequencies to construct a network-level measure of edge variability defined as:

$$\begin{array}{c} S^{k}=1-4\frac{1}{q}\sum_{e}f_{e}^{k}\left(1-f_{e}^{k}\right) \end{array}$$

where *q* = *p*(*p* − 1)/2 is the total number of possible edges and *S*^*k*^ can be thought of as the mean of (edge-level) Bernoulli variances. Each value λ_*k*_ is associated to a single selection frequency vector, and a resulting stability value. Finding the right edge frequency is therefore equivalent to finding the right stability level. Classical choices for stability are *stab* = 80% or *stab* = 90%, as suggested in [24], to have a good compromise between recall and precision and the optimal level λ^*^ is chosen as $\lambda^{*}=\underset{\lambda }{\text{min}}\;S\left(\lambda \right)\geq stab$. Once the optimal level λ^*^ is fixed, it is common practice to refit the model by running the inference method on the full dataset *X* with the λ^*^ chosen by StARS, to return the corresponding graph.

#### Using edge-level selection frequencies rather than network-level stability

We present here our suggested modification of the StARS algorithm allowing us to use edge-level frequencies to build consensus networks. Instead of computing the network-level stability *S*^*k*^ and doing a final refit step, as is done in StARS, we instead select directly edges with high selection frequency to create the set $E^{\lambda }\left(c\right)=\left\{e,\;\;f_{e}^{\lambda }>c\right\}$ of highly reproducible edges, where *c* is a constant close to 1, typically 0.9 or higher. In this way, we guarantee both high precision and high reproducibility for edges in *E*^λ^(*c*) as they are selected many times in the resampling. Smaller values of λ give rise to larger sets *E*^λ^(*c*) and higher recalls. Two advantages of using frequencies rather than refitting the network are (i) filtering out edges with low support that could be included in the refit graph and (ii) making it easier to combine the edges inferred by the different methods.

### Step 2: Select λ to equalize of the densities of the networks

In order to include the best edges in the consensus network, we must choose one λ per method. A natural choice would be the value $\lambda_{m}^{⋆}$ selected by StARS for the method *m*. However, we observed that StARS computes a very different precision/recall for each method as it does not integrate the same number of edges in the graph (S1 Fig). We select instead the smallest λ_*m*_ such that (i) the sets $E_{m}^{\lambda_{m}}\left(c\right)$ are roughly of equal sizes and (ii) the mean stability is above a given threshold: $\frac{1}{M}\sum_{m=1}^{M}S_{m}\left(\lambda_{m}\right)\geq stab$. It forces all methods to contribute with a similar number of edges to the consensus while ensuring that each edge set is reproducible. In the following, we applied the mean-stability with a coefficient of 90% as mentioned in the previous subsection. In practice, to match edge set sizes, we worked with edge density rather than with λ values as the two are monotonically related.

### Step 3: Summarize the sets of selection frequencies across methods

Building a consensus network from the sets of edges *E*^λ^(*c*) produced by the different methods is akin to an ensemble procedure where many methods are combined together.

In order to produce a stable and accurate consensus network, we define several summary metrics aimed at mitigating the drawbacks of each method while benefiting from their strengths. The consensus is obtained by summarizing edges frequencies across the methods. Denoting by *f*_*m*_ the selection frequency of a given edge with method *m* ∈ {1, …, *M*}, we define:

- *mean*: average selection frequency ∑_*m*_ *f_m_*/*M*,
- *norm2*: euclidean norm (2-norm) ${\left(\sum_{m}f_{m}^{2}\right)}^{1/2}/M^{1/2}$,
- *IVW*: inverse-variance weighted average $\left(\sum_{m}f_{m}\times \frac{1}{f_{m}\left(1-f_{m}\right)})/(\sum_{m}\frac{1}{f_{m}\left(1-f_{m}\right)}\right)$, where *f*_*m*_ follows a Bernoulli with $\hat{Var}\left(f_{m}\right)=f_{m}\left(1-f_{m}\right)$,
- *minp*: high frequency for at least *p* methods $1_{\left\{\sum_{m}\left(f_{m}>c\right)\geq p\right\}}$.

We chose the mean as it is the most widely used metric to summarize a set of value, and *minp* as it is common practice in ensemble methods to keep a variable selected by at least *p* methods in the ensemble. Norm2 skews the summary towards high frequencies whereas Inverse-Variance Weighted (IVW) average upweights high and low frequencies as they are easier to estimate than mid-range frequencies. All summaries except *minp* can account for method-specific weights *w*_*m*_ by replacing *f*_*m*_ (resp. *M*) with *w*_*m*_*f*_*m*_ (resp. ∑_*m*_*w*_*m*_). In this work, we only considered the simple case *w*_*m*_ = 1. Finally, note that *minp* is the only one that returns a binary summary, all the other ones take value in [0, 1], like the original selection frequencies.

To reinforce the methodological assessment of OneNet, we also considered a naive consensus, called hereafter MRC for Majority-Rule Consensus, similar in spirit to min4 but applied directly to the refit networks, rather than to edge selection frequencies. MRC simply consists in running all methods with their default settings (*e.g.* without aiming for similar number of edges in all networks) and keeping only edges inferred in at least half the networks. Note that MRC and the different variants of OneNet have almost identical computational costs as all inference methods, but COZINE already include a StARS step.

### Statistical analyses

All methods were compared in terms of PPV using a one-way ANOVA followed by Tukey’s HSD post-hoc test to assess pairwise significant differences. On the boxplot figures, results are shown using the compact letter display: two methods are significantly different if they don’t share a common letter and small letters (*e.g.* “a”) correspond to methods with higher PPV. Boxplots use the following convention: the box ranges from the first (Q1) to the third (Q3) quartile while the median (Q2) corresponds to the line within the box, whiskers extends 1.5 × (Q3—Q1) below and above the box and all points outside the whiskers are considered outliers and shown as dots.

### Datasets

#### Simulated dataset

In this work we simulate data using the methodology described in [13] which is based on gaussian copula to control the network structure followed by sampling from the species marginal distributions to preserve the peculiarities of abundance data. This method yields synthetic data with marginal distributions that are closer to the original empirical dataset, while enforcing a given correlation structure between the species.

To generate the simulated dataset, we use in input the empirical dataset described below restricted to diseased individuals and transform the abundance table into count table.

The dataset is simulated in the framework of an unknown undirected graph *G*(*V*, *E*), with no retroactive loop, consisting of *p* vertices *V* = {1, …, *p*} and a set of edges *E* ⊆ *V* × *V* connecting each pairs of vertices. The graph G is represented by its adjacency matrix *A* = (*A*_*ij*_)_(*i*, *j*) ∈ *E*_ of size *p* × *p*, defined as:

$$\forall \left(i,j\right)\in ⟦1,p{⟧}^{2},\;\;A_{ij}=\{\begin{array}{c} 1\;\;\text{if}\;(i,j)\in E,\\ 0\;\;\text{otherwise.} \end{array}$$

The package EMtree v.1.1.0 [25] is used to generate a precision matrix *Ω* defined as the graphical Laplacian *A* of a cluster graph. *Ω* is inverted to create the correlation matrix Σ and the idea was then to simulate variables with arbitrary marginal distributions from multivariate normal variables with correlation structure given by Σ using gaussian copula. Specifically, we generate a *n* × *p* matrix *Z* with independent normal rows $Z_{i}∼N\left(0,\Sigma \right)$. We then get uniform random vectors by applying standard normal cdf transformation to each column of *Z*, $u^{j}=\phi \left(Z^{j}/\sqrt{\Sigma_{jj}}\right)$ element-wise, and finally apply the quantile functions of the empirical data marginal distributions to each *u*^*j*^. The function synthData_from_ecdf from the SPRING package v.1.0.4 [26] is used for these simulation steps. To assess the effect of sample size, we simulate datasets of size *n* ∈ {50, 100, 500, 1000}.

### Evaluation criteria

Each method is evaluated by comparing the inferred network structure to the known simulated network structure using the following metrics:

- Precision (positive predictive value): PPV = TP/(TP+FP),
- Recall (true positive rate): TPR = TP/(TP+FN),

where TP stands for True Positive (a correctly detected edge), FP for False Positive (an edge detected where none should be) and FN for False negative (an undetected edge). The precision measures the proportion of real edges among the detected ones, whereas the recall measures the proportion of real edges which are detected.

#### Empirical dataset

The empirical dataset, studied in [27], corresponds to stool samples from 216 Chinese individuals sequenced using whole-metagenome sequencing techniques. The raw sequences are available as BioProject PRJEB6337 in the European Nucleotide Archive (ENA). Among this population, 102 individuals are healthy and 114 suffer from liver cirrhosis. Abundances of all microbial species (metagenomic species or MSP) detected using 10.4 million IGC2 gut gene catalogue [28] are extracted using the Meteor software suite that creates a gene abundance table by mapping high quality reads onto the gene catalogue, using Bowtie2. Abundance of each MSP is computed as the mean abundance of 100 marker genes selected for that MSP, where the gene abundance is the read abundance normalized by the gene length. The abundance table is then transformed into a count table of size 1990 MSP by 216 individuals [29]. In the Application section, we used only the 114 cirrhotic patients.

## Results

In this section we evaluated the performances of both OneNet and the network inference methods on the simulated dataset.

### Influence of the stability level on the inferred graphs

We first evaluated the effect of stability level on the performance of the inference methods. Instead of fixing a target stability at 0.8 or 0.9, we studied the relationship between the precision and recall of the inferred edges by each method for different stability levels. Because interactions between highly prevalent species are easier to reconstruct, we only kept metagenomic species with a prevalence greater than 50% (159 species). We let the sample size vary in {50, 100, 500, 1000} and we considered *B* = 40 resamples each time.

#### Methods have distinct precisions for a given stability level

S4 Fig shows the relationship between the performance obtained with the edge set $E^{\lambda^{*}}\left(0.90\right)$ (precision PPV90 and recall TPR90), and the corresponding stability. The difference in patterns grows with sample size *n*, revealing peculiarities inherent to each method. Clearly, methods have distinct performances for the same stability level. We observed that methods cluster in groups (glasso, neighborhood selection, tree aggregation) with different precision/recall tradeoffs. As a result, they produce edge sets that greatly differ both in size and quality. This suggests that the stability value is not a good indicator of the precision level achieved by each method.

#### Methods have comparable precision and recall for a given density level

Unlike precision, which is unavailable when dealing with empirical datasets, the density, or number of detected edges, can always be computed. S5 Fig shows the relationship between precision (resp. recall) and density for all methods at increasing sample sizes. The curves are almost superimposed for values of *n* up to 100, after which different behaviors appeared. However, the gap in performance between methods stayed small when imposing the same density, rather than the same stability. This also meant that, whatever the method used, the *m* first edges included in the graph achieve similar graph reconstruction quality, although they correspond to different stabilities. We thus selected individual graphs based on density, rather than stability, to include only graphs with similar precision and recall in the consensus phase.

#### Mean stability as a proxy of the density level

The previous observation prompted us to explore the link between density and stability for different values of λ. S6 Fig shows how stability decreases with increasing density. We set a target mean stability value (e.g. 90%) for each value of *n* (here between 50 and 1000). As *n* grows, we observed that the density increased from 90 to 170, as well as the spread between methods. For *n* = 1000, stabilities ranged from 0.8 for EMtree to 1 for SPRING. We can see how targeting the mean stability rather than the same stability for all methods allows to adapt the precision level of each method through density to make them more similar.

#### Mean stability increases the consistency between the performances of the network inference methods

**Fig 3:**
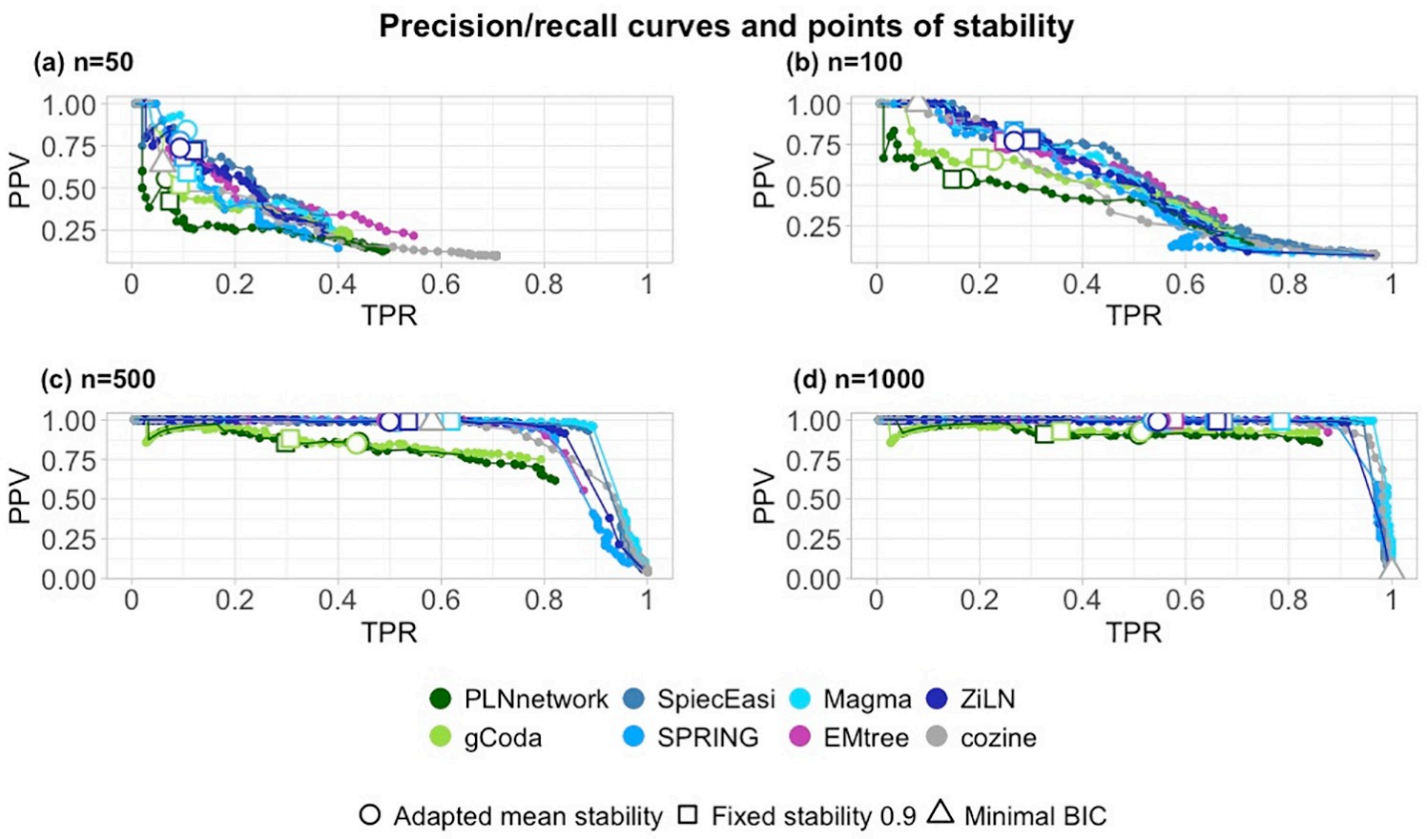
Precision—Recall curves of each inference method for different sample sizes. (a) 50 (b) 100 (c) 500 (d) 1000. The TPR/PPV compromise achieved for λ^*^ corresponding to a stability of 0.9 is shown with a circle, the one achieved by a mean stability across methods of 0.9 is shown with a square. Whenever the selected λ is the same, the circle and the square are replaced with a diamond. Finally, note that COZINE relies on minimization of a BIC criteria (shown with a triangle) rather than on the resampling-based stability selection to choose the regularization parameter.

We compared, for different sample sizes, the precision—recall tradeoff achieved by the mean stability to the ones achieved by a fixed stability (e.g. 0.9). These sizes are typical of datasets used in real applications and in our motivating example. They also correspond to sample sizes frequently used in the literature of microbial reconstruction networks. For each of the sizes, we simulated one dataset and saved one graph per value of λ. The precision and recall values are calculated from each inferred graph. Fig 3 shows that the sample size has a major impact on the precision and stability. The ROC curves stabilize to near-perfection starting from *n* = 500 (Fig 3c). It is also noticeable that the adapted stabilities reduced the range of the method’s precision. Furthermore, for glasso-based methods (gCoda, PLNnetwork), the new target led to a 20 points improvement in TPR at almost no cost in PPV, for large sample sizes. Note that COZINE performs especially poorly compared to other methods for large sample sizes (*n* = 1000, Fig 3d), with a TPR close to 1 but a PPV close to 0. This is due exclusively to the use of a fast BIC critera instead of the resampling-based StARS which selects a small λ leading to a very dense graph in this example. Very poor performances of COZINE are also observed on large sample sizes (*n* ≥ 500, S7 and S8 Figs) but not on small ones (*n* = 100, S9 Fig).

### OneNet versus the classical network inference methods

**Fig 4:**
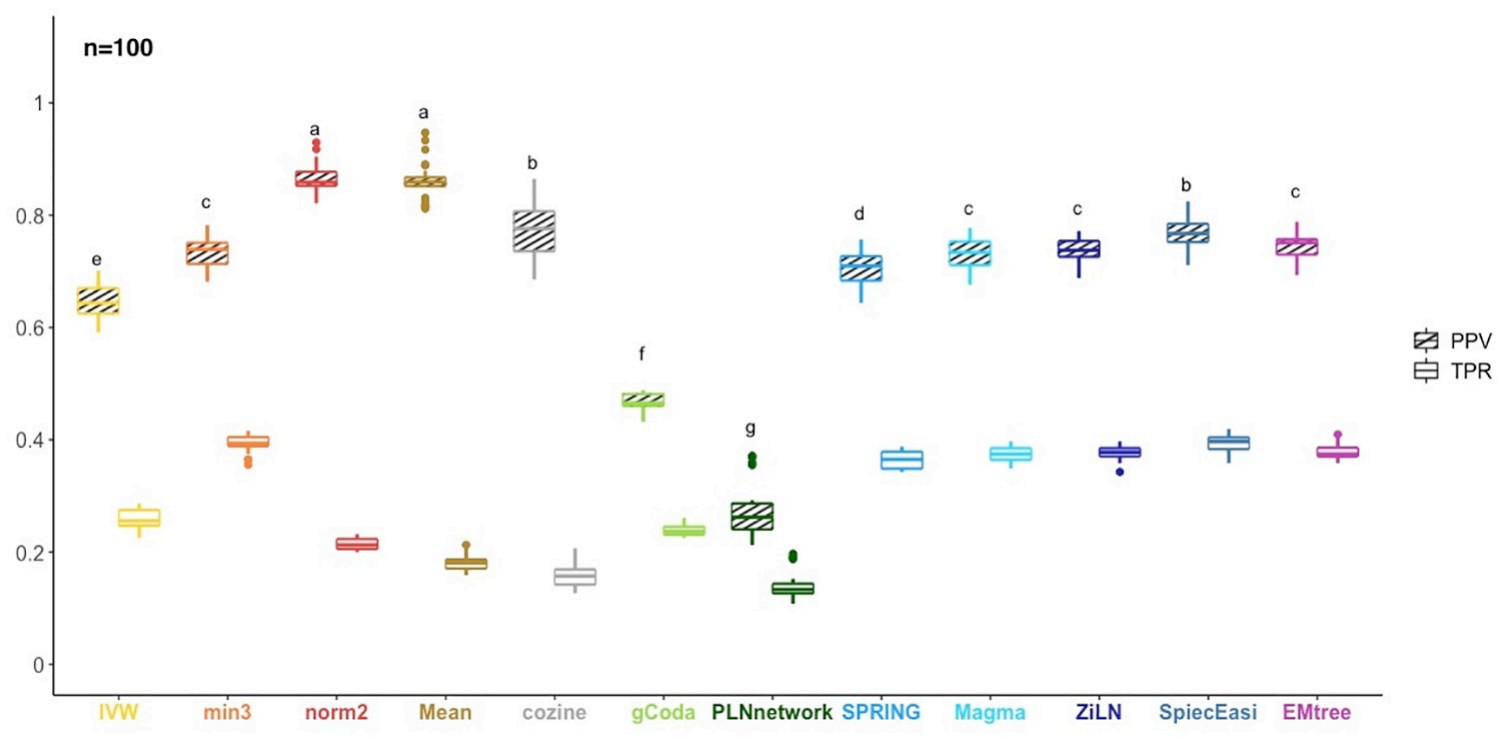
Quality of both single-method and consensus network in terms of PPV/TPR assessed on 20 simulated datasets of size *n* = 100 samples. Striped (resp. no-strip) boxplots show PPV (resp. TPR) values.

We computed, for a frequency threshold of 90%, the precision and recall values obtained by the classical network inference methods (COZINE, gCoda, PLNnetwork, SPRING, Magma, SpiecEasi, ZiLN and EMtree) on 20 simulated datasets and we compared them to the OneNet networks (with the summary metrics mean, norm2, IVW and min3). For all individual methods, we used fixed stability (0.9) to choose λ and the edge sets while for the consensus ones, we used mean stability criteria (0.9) to harmonize the density of individual networks before computing the consensus. Note that because of the similar density, each method provided roughly the same number of edges to OneNet. Fig 4 shows that OneNet with the mean and norm2 consensus methods, achieved the best precision levels (one-way ANOVA followed by Tukey’s HSD post-hoc test, *p* < 2.10^−16^) but the worse recall values. OneNet with the min3 summary has comparatively lower precision but higher recall and OneNet with the IVW summary has both worse recall and worse precision. This illustrates how OneNet generally leads to sparser networks with higher precision.

We also observed that the MB-based methods tend to outperform glasso-based ones. However, OneNet still demonstrated precision levels equivalent to the best methods when including (S2 Fig) or not (S3 Fig) the methods based on graphical lasso. This reflects the inherent robustness of consensus measures to methods with outlying performance.

On top of that, sample size *n* has a dramatic effect on all criteria and methods. For large sample sizes (*n* ≥ 500), most methods exhibit precision above 99% and recall between 30% and 60% (S2 and S3 Figs, panels (c) and (d)). By contrast (S2(a) and S3(b) Figs) for smaller but more realistic sample sizes like *n* = 50 (resp. *n* = 100), the median precision drops below 60% (resp. 80%) for all methods except OneNet-Mean and OneNet-norm2 which both remain above 75% (resp. 85%). Likewise, the recall drops below 40% for *n* = 100 (S3(b) Fig) and below 20% for *n* = 50 (S3(a) Fig).

We observed a discrepancy in terms of precision and recall for the COZINE method between Fig 3 and S3 Fig. We hypothesized that it’s due to the original COZINE procedure (BIC criteria) used to select the optimal network, which leads to a dense graph (very high recall, very low precision, and therefore many spurious edges, see Fig 3). By contrast, S3 Fig shows the precision and recall values obtained with the resampling approach applied to COZINE are in line with other methods. This is an extreme example of lack of robustness, where the network reconstructed from the full dataset differs drastically from the ones reconstructed on random subsets of the data and illustrates the benefits of combining the resamples rather than doing a refit. Note that the difference between refit and combined resamples is much smaller for other methods as they already include a stability selection step designed to mitigate those problems. To explore further the benefits of combining edge selection frequencies rather than edges in the refit models, we compared OneNet variants to the MRC consensus (S10 Fig). We observed that MRC achieves a slightly higher TPR but a slightly lower PPV than OneNet-mean and OneNet-norm2 on synthetic data and thus constitutes a relevant approach.

Finally, the upset plots of edges detected by the inference methods for *n* = 100 (S11 Fig) and *n* = 500 (S12 Fig) show the same trends: (i) a large, but decreasing with *n*, number of edges undetected by any method, (ii) most reconstructed edges (whether wrongfully or not) detected by many methods and (iii) very few edges detected by only one or two methods (with the exception of gCoda and PLNnetwork). This strong agreement between all methods and their overall very high precision on large datasets (*n* > 500) is likely a consequence of our semi-parametric simulation setting and may constitute an optimistic evaluation of the methods.

## Application to liver cirrhosis

To investigate the performance of OneNet relative to the other methods, we inferred all the networks from the microbiome dataset of cirrhotic patients presented in the Methods [27]. Because of the small size of the dataset (114 samples), only metagenomic species with a prevalence greater than 50% were kept (155 species). The networks have been clustered using the CORE-clustering algorithm to reconstruct microbial guilds [30]. Following the guidelines of this paper, we fixed the number of clusters between 10 and 19.

**Fig 5:**
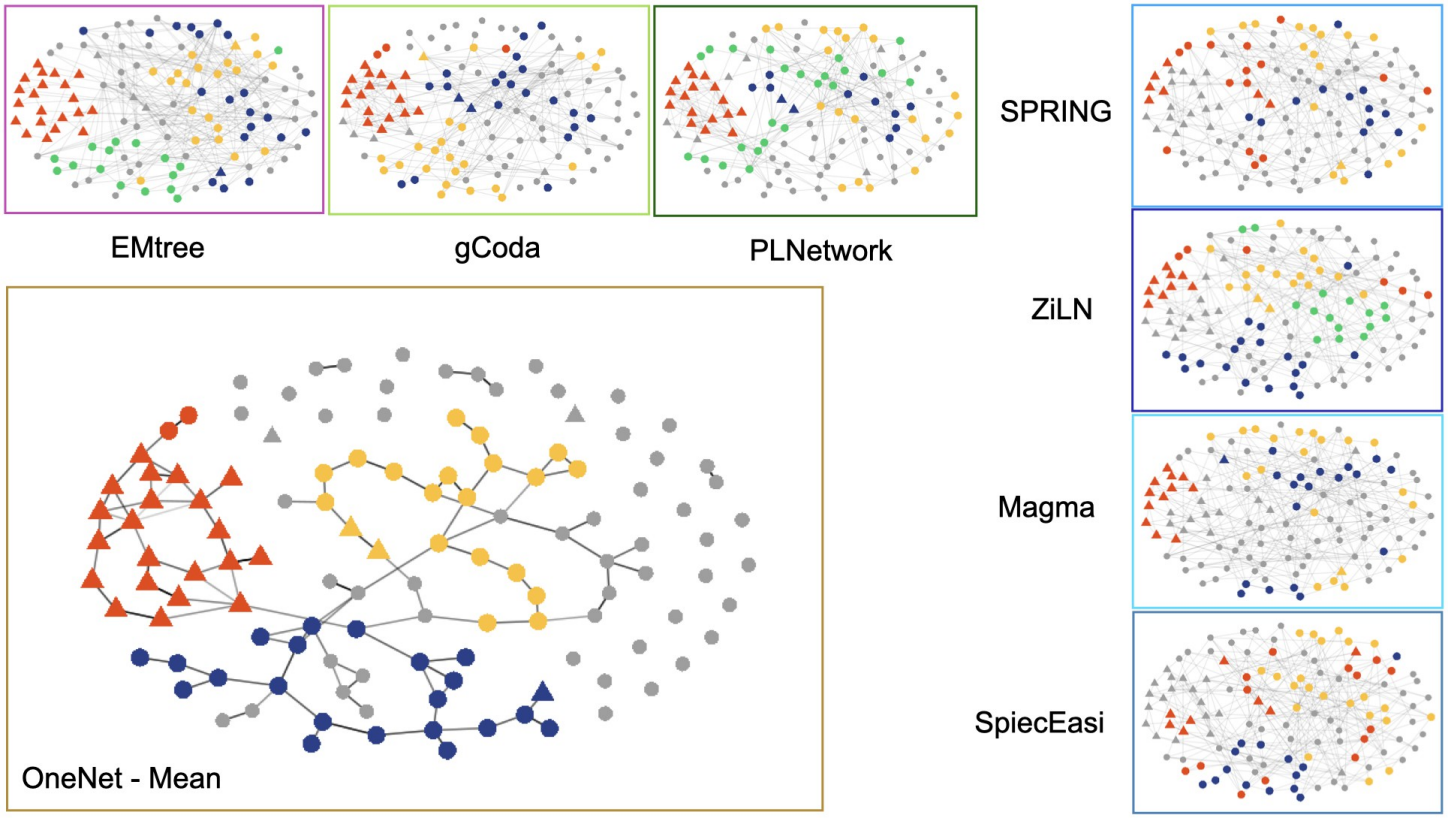
Consensus and single-method networks inferred on the liver cirrhosis dataset, followed by CORE-clustering algorithm to identify the microbial guilds. All graphs share the same layout, computed on the OneNet-mean network, to ease comparisons. Nodes are colored by cluster, with red always used for the cirrhotic guild in all graphs where it is (at least partially) recovered and species prevalent in the oral cavity are represented by a triangle. Methods are grouped based on the underlying inference technique (tree aggregation, graphical lasso, neighborhood selection).

Fig 5 illustrates the inferred and clustered networks. We note that the OneNet-mean network is the sparsest one, consistent with our results on the simulated datasets (lower TPR but higher PPV as shown in Fig 4 and sparser graphs as shown in the upset plots S11 and S12 Figs). Applying CORE-clustering to each network always resulted in 3 to 4 guilds.

**Fig 6:**
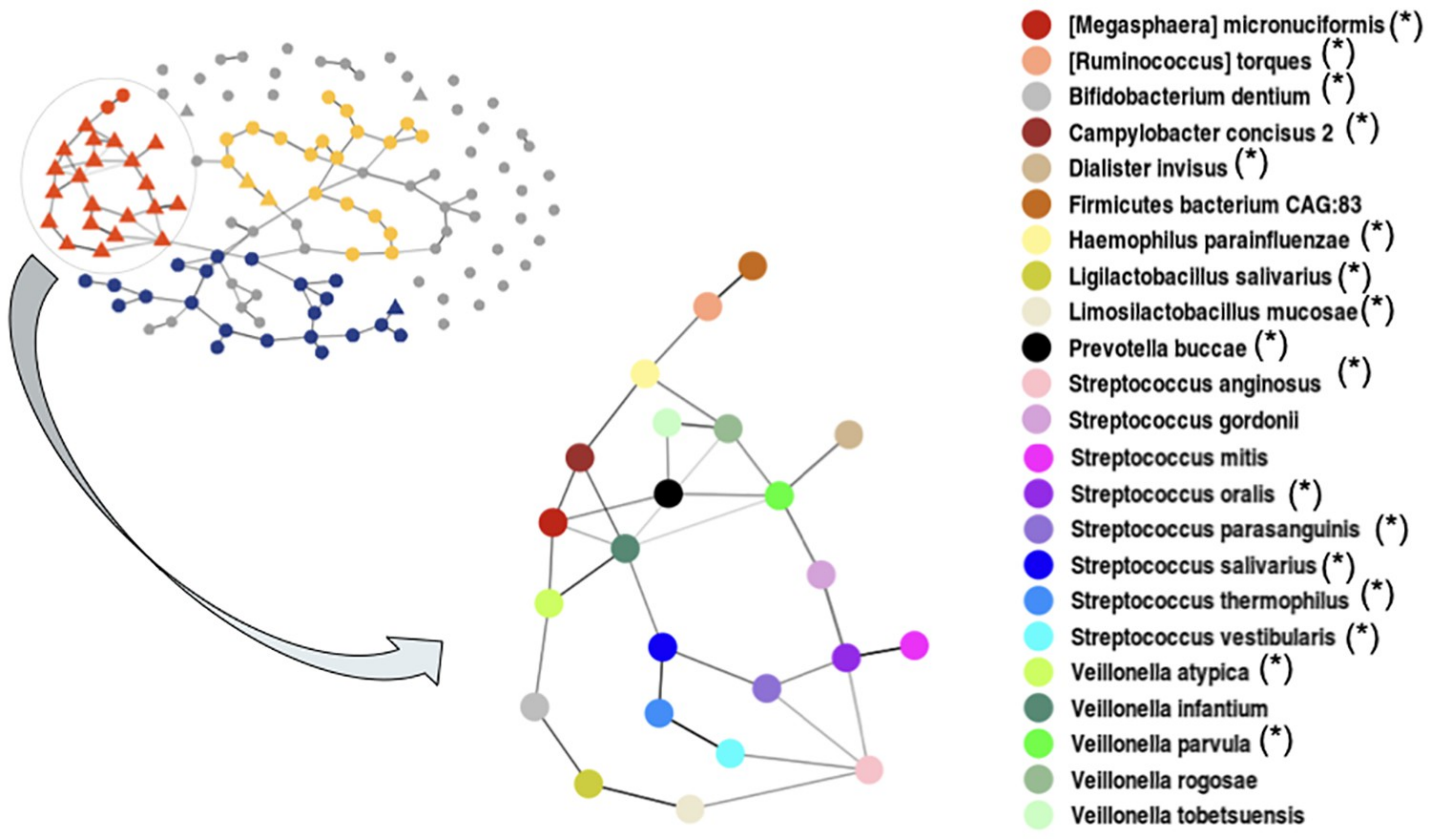
Detailed view of the cirrhotic guild identified in the OneNet-mean network with taxonomic information on the nodes. All species known to be associated with chronic diseases are marked with a star (*).

One guild—the “cirrhotic guild”, shown in red in Fig 5 and highlighted in Fig 6, is notable as it contains species associated to chronic diseases: obesity after weight-loss intervention (OB after WL) [31], schizophrenia (SCHIZO) [32]), atherosclerotic cardiovascular disease (AVCD) [33], Crohn disease (CROHN) [34] and liver cirrhosis (LIVER) [27] (S1 Table). Interestingly, the majority of the species detected in the cirrhotic guild, represented by a triangle shape, are prevalent in the oral cavity. This result fits with [27], which highlights the invasion of species prevalent in the oral cavity in liver cirrhosis.

Among the 23 identified species of the cirrhotic guild, 17 have been found associated together in networks of chronic diseases (see S1 Table). These results are therefore consistent with what has already been shown in the literature.

Among the different methods included in the consensus, only some (Magma, gCoda, PLNnetwork, EMtree, ZiLN) were able to recover (at least partially) the cirrhotic guild from the consensus (highlighted in red in Fig 5). The upset plot S13 Fig also shows greater disparities between methods and greater differences between norm2- and mean-consensus (with mean being the more restrictive) than expected from the synthetic data. In particular, some neighborhood selection methods (SPRING, SpiecEasi, Magma) and EMtree have a lot of unique edges (S13 Fig), in contrast with their behavior on synthetic data (S11 and S12 Figs) suggesting that they react differently to model misspecifications. Note that all edges selected by the mean-consensus are selected in at least 5 (out of 7) individual methods showing that mean-consensus is more stringent than a simple majority rule.

By contrast, the network and microbial guilds reconstructed from the healthy individuals (*n* = 102 individuals and *p* = 151 taxa with prevalence higher than 50%) using the same parameters exhibited a completely different structure (S14 Fig). While we recovered the same number (3) of guilds, none matched the cirrhotic microbial guild. Most taxa from the the cirrhotic guild were either not present in the “healthy individuals”-network (as they didn’t pass the prevalence threshold in the healthy individuals dataset) or had much fewer interactions and were not included in a guild. Overall, this suggests that the interactions identified between the cirrhotic species are context-dependent, a finding that has already been documented in other contexts [35] and [36].

## Discussion

The proposed framework, with a microbial consensus network inference method, offers new insights about inferring robust and sparse microbial networks. OneNet is robust in the sense that i) it uses GGM adapted to deal with the peculiarities of microbial abundance properties (inclusion of environmental effects as covariates, stabilization of data variability, adaptation to abundances with high proportion of zeros, etc), ii) it depends on seven network inference methods aiming for sparse and reproducible microbial network using either glasso, neighborhood selection or tree averaging approaches, iii) it relies on a three-step procedure to improve the precision and reproducibility of both inference methods and OneNet. Indeed, the selection of edges with high inclusion frequencies and harmonization of stability selection achieve similar precision levels across methods The resulting consensus network uses a summary of the edge inclusion frequencies.

Results from the studies on synthetic and real data illustrated the first major and reassuring fact, that the inference methods overall agree with one another and with a fraction of the truth (S11 and S12 Figs), the remainder being hard-to-reconstruct edges. It then showed the effectiveness of OneNet compared to the inference methods. Among the different summaries considered, the mean or norm2 are preferred since they lead to slightly sparser networks but achieved much higher precision than any inference method, especially for sample sizes around *n* = 100, which is typical in microbiome studies. By contrast, min3 and IVW summaries gave a significant additional quantity of edges compared to the other summary metrics, yielding TPR levels that are comparable to those obtained with glasso-based methods without increasing the PPV, especially when the number of samples is small (*n* ≤ 100) (S13 Fig).

In all numerical experiments, we showed that a minimal sample size to maintain high robustness was *n* = 100. In this scenario we suggest to use the mean summary in OneNet, as it proved to be more robust to small sample sizes. Obviously, the precision is affected by both the number of samples and microbial species in the system, the latter being controlled by the prevalence threshold imposed at the very beginning of the analysis. As illustrated on S7, S8 and S9 Figs, the prevalence threshold can be adjusted to increase the precision of the method depending on the number of samples (*prev* = 0.50 for *n* = 100 and *n* = 500, *prev* = 0.20 for *n* = 1000). From *n* = 1000, when considered individually, the neighborhood selection and the tree averaging approaches showed performances that were similar to OneNet. In this context, it could be possible to select one of these three approaches.

We also investigated a simple alternative (MRC) based on majority rule for edge selection. This simple alternative was competitive to OneNet variants on synthetic data, with slightly lower PPV and slightly higher TPR. We however observed that the inference methods produces results that were more contrasted in terms of edge sets on real data than on synthetic data (compare S11 and S13 Figs). It means that inference methods react differently to model misspecification in real life settings and may have lower TPR and PPV than estimated from the simulations and that some methods may contribute with a large number of spurious edges to the consensus. This was for example the case of COZINE for large sample sizes in our simulations. While this shortcoming also applies in principle to OneNet variants, it is mitigated by using roughly the same number of edges from each method (step 2 of the algorithm). Finally, MRC is sensitive to methods which can fail drastically, as COZINE, whereas the compulsory resampling step used in OneNet stabilizes the results of each methods and prevents catastrophic behavior. This is less of an issue if all methods of the consensus use stability selection but then the computational overhead of MRC and OneNet variants are comparable. Overall, both OneNet and MRC perform better than no consensus but, compared to MRC, OneNet(-mean) is more robust than MRC and favors PPV to TPR.

An advantage of OneNet is its ability to easily incorporate new inference methods as soon as they are amenable to the modified stability selection framework used here. This is the case for all the methods considered in this work but COZINE. Indeed, COZINE relies on the BIC criteria to tune the regularization parameter λ: it doesn’t allow the user to provide a fixed λ grid for comparisons with other methods and doesn’t produce the table of edge selection frequencies required to compute summaries. This is however due to implementation choices (using BIC instead of StARS for selecting λ) rather than to fundamental incompatibilities with the OneNet framework.

## Supporting information

S1 Table Among the 23 identified species of the cirrhotic guild, 17 have been found associated together in networks of chronic diseases. The Species column lists these 17 species. Each column named after a chronic disease corresponds to a microbial network shown in the referenced article.
(PDF)

S1 Fig Precision—Recall curves of each inference method before equalizing the densities and TPR/PPV value obtained for λ^*^ corresponding to default stability criteria shown with a square (*n* = 100). All method have a distinct TPR/PPV compromise and don’t select the same number of edges in the graph.
(TIF)

S2 Fig Compared precision (PPV) and recall (TPR) of inference methods and OneNet-* variants when including all 7 methods in the set of methods, for different samples sizes. (a) *n* = 50 (b) *n* = 100 (c) *n* = 500 (d) *n* = 1000.
(TIF)

S3 Fig Compared precision (PPV) and recall (TPR) of inference methods and OneNet-* variants after removing glasso-based methods from the set of methods, for different samples sizes. (a) *n* = 50 (b) *n* = 100 (c) *n* = 500 (d) *n* = 1000.
(TIF)

S4 Fig PPV—Stability and TPR—Stability curves of the edge set *E*^λ^(0.90) according to the sample size and inference method. Each point in the curve corresponds to a different value of λ.
(TIF)

S5 Fig PPV—Density and TPR—Density curves of the edge set *E*^λ^(0.90) according to the sample size and inference method. Each point in the curve corresponds to a different value of λ.
(TIF)

S6 Fig Stability—Density curves of the edge set *E*^λ^(0.90) according to the sample size and inference method. Each point in the curve corresponds to a different value of λ. The grey dashed horizontal line represents the target mean stability value (0.90) and the black vertical one, the associated density.
(TIF)

S7 Fig Precision—Recall curves of each method and TPR/PPV compromise chosen by stability, mean stability and BIC when *n* = 500 after filtering the dataset to keep only species with prevalence higher than a given threshold. (a)0.20 (b)0.50 (c)0.8 (d)0.9 (see Fig 3 for details).
(TIF)

S8 Fig Precision—Recall curves of each method and TPR/PPV compromise chosen by stability, mean stability and BIC when *n* = 1000 after filtering the dataset to keep only species with prevalence higher than a given threshold. (a)0.20 (b)0.50 (c)0.8 (d)0.9 (see Fig 3 for details).
(TIF)

S9 Fig Precision—Recall curves of each method and TPR/PPV compromise chosen by stability, mean stability and BIC when *n* = 100 after filtering the dataset to keep only species with prevalence higher than a given threshold. (a)0.20 (b)0.50 (c)0.8 (d)0.9 (see Fig 3 for details).
(TIF)

S10 Fig Quality of consensus networks (MRC and OneNet consensus) in terms of PPV/TPR assessed on simulated datasets of size *n* = 100 samples. Striped (resp. no-strip) boxplots show PPV (resp. TPR) values.
(TIF)

S11 Fig Upset plot of the edges identified by the inference methods, the OneNet-* variants and the ground truth on the synthetic dataset for *n* = 100. (TIF)

S12 Fig Upset plot of the edges identified by the inference methods, the OneNet-* variants and the ground truth on the synthetic dataset for *n* = 500. (TIF)

S13 Fig Upset plots of the edges identified by the inference methods and the OneNet-* variants applied to the liver cirrhosis dataset. (TIF)

S14 Fig Consensus network inferred on the healthy individuals (n = 102 samples, p = 151 species) from the liver cirrhosis dataset, followed by CORE-clustering algorithm to identify the microbial guilds. The guilds are represented by *, ∘ and □ and species from the cirrhotic guild are highlighted in color using the same color code as in Fig 6.
(TIF)

S1 File Supplementary methods. (PDF)
