# Supplementary material for: OneNet—One network to rule them all: Consensus network inference from microbiome data
Source: PLoS Comput Biol. 2024 Dec 6;20(12):e1012627. doi: 10.1371/journal.pcbi.1012627 (PMC11654977; doi:10.1371/journal.pcbi.1012627)
Supplement: S1 Table — The Species column lists these 17 species. Each column named after a chronic disease corresponds to a microbial network shown in the referenced article. (PDF) [file pcbi.1012627.s001.pdf]

S1 Tab. Species of the cirrhotic guild associated with chronic diseases.

Species	LIVER [1]	SCHIZO [3]	OB after WL [2]	AVCD [4]	CROHN [5]
[Megasphaera] micronuciformis	x	x	x	x	
[Ruminococcus] torques			x		x
Bifidobacterium dentium	x	x		x	
Campylobacter concisus 2	x	x		x	
Dialister invisus		x		x	
Haemophilus parainfluenzae	x				
Ligilactobacillus salivarius (ex Lactobacillus)	x			x	
Limosilactobacillus mucosae (ex Lactobacillus)	x			x	
Prevotella buccae	x			x	
Streptococcus anginosus	x	x		x	
Streptococcus oralis	x				
Streptococcus parasanguinis	x			x	
Streptococcus salivarius	x	x			x
Streptococcus thermophilus				x	
Streptococcus vestibularis	x			x	
Veillonella atypica	x	x			
Veillonella parvula	x	x			

Among the 23 identified species of the cirrhotic guild, 17 have been found associated together in networks of chronic diseases. The Species column lists these 17 species. Each column named after a chronic disease corresponds to a microbial network shown in the referenced article.

References

1. , Qin N, and al. Alterations of the Human Gut Microbiome in Liver Cirrhosis. Nature. 2014 513(7516), pp.59-64.

2. , Liu R, and al. Gut microbiome and serum metabolome alterations in obesity and after weight-loss intervention. Nature Medicine. 2017 23(7), pp.859–868.

3. , Zhu F, and al. Metagenome-wide association of gut microbiome features for schizophrenia. Nature Communications. 2020 11(1612).

4. , Jie Z, and al. The gut microbiome in atherosclerotic cardiovascular disease. Nature Communications. 2017 845(8).

5. , He Q, and al. Two distinct metacommunities characterize the gut microbiota in Crohn's disease patients. Nature Communications. 2017 6, pp.1–11.
