# Supplementary figures and images for: OneNet—One network to rule them all: Consensus network inference from microbiome data
Source: PLoS Comput Biol. 2024 Dec 6;20(12):e1012627. doi: 10.1371/journal.pcbi.1012627 (PMC11654977; doi:10.1371/journal.pcbi.1012627)

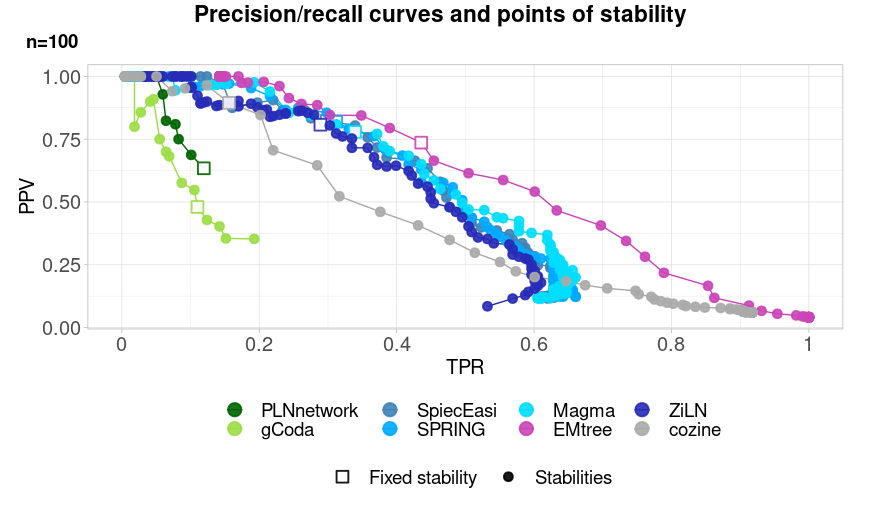

Supplement: S1 Fig — All method have a distinct TPR/PPV compromise and don’t select the same number of edges in the graph. (TIF) [file pcbi.1012627.s002.tif]

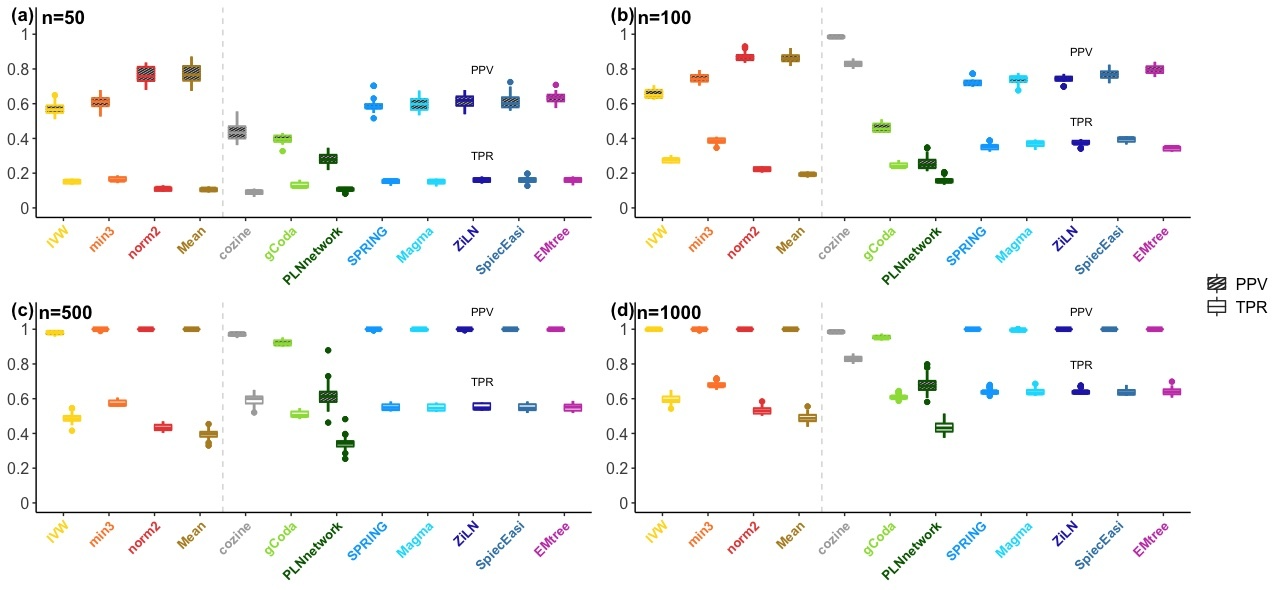

Supplement: S2 Fig — (a) n = 50 (b) n = 100 (c) n = 500 (d) n = 1000. (TIF) [file pcbi.1012627.s003.tif]

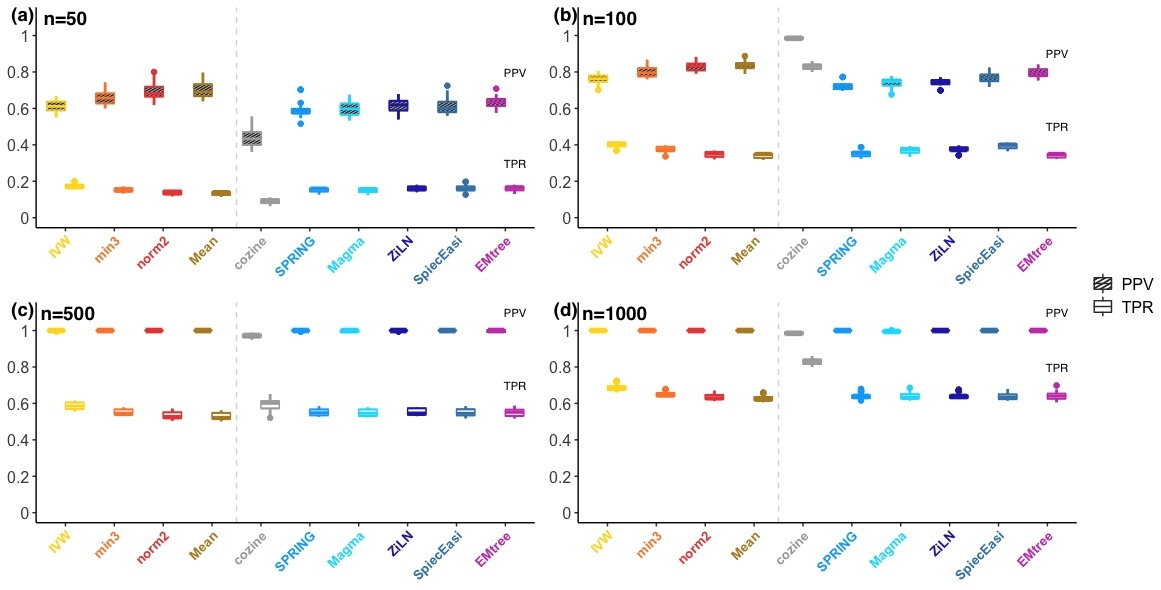

Supplement: S3 Fig — (a) n = 50 (b) n = 100 (c) n = 500 (d) n = 1000. (TIF) [file pcbi.1012627.s004.tif]

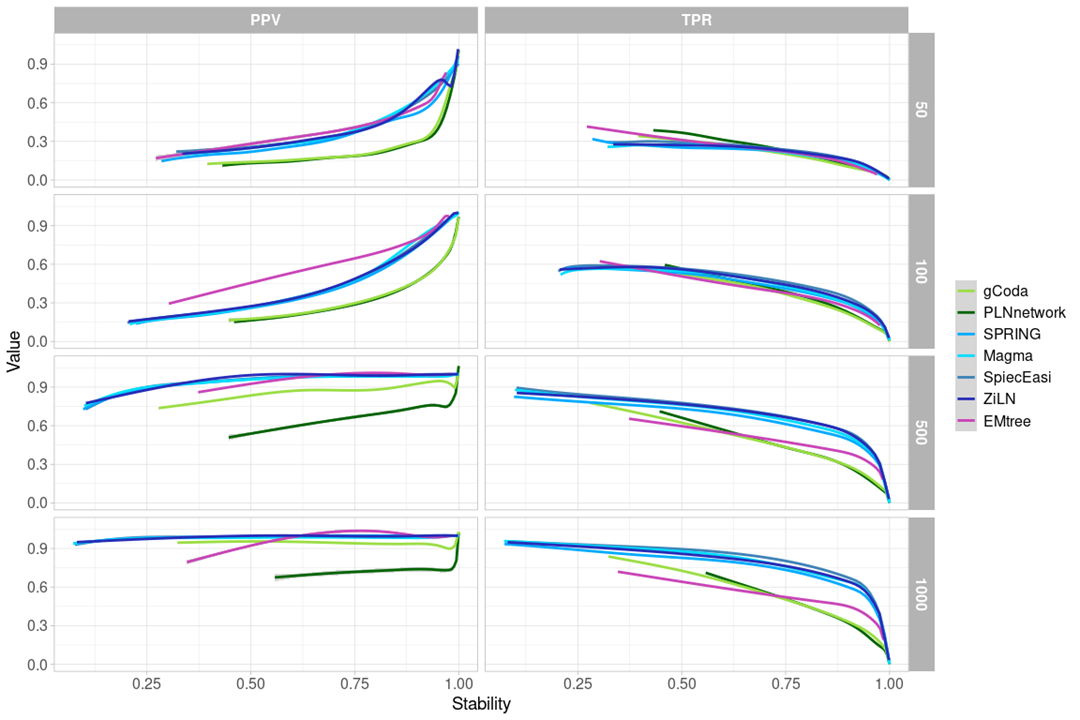

Supplement: S4 Fig — Each point in the curve corresponds to a different value of λ. (TIF) [file pcbi.1012627.s005.tif]

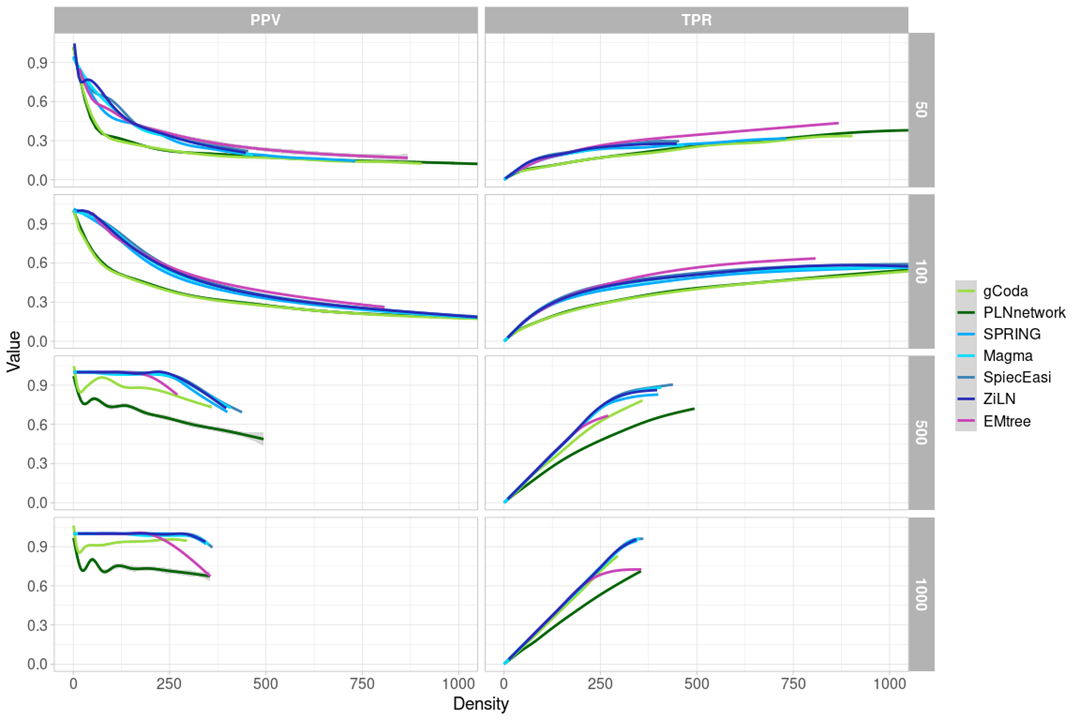

Supplement: S5 Fig — Each point in the curve corresponds to a different value of λ. (TIF) [file pcbi.1012627.s006.tif]

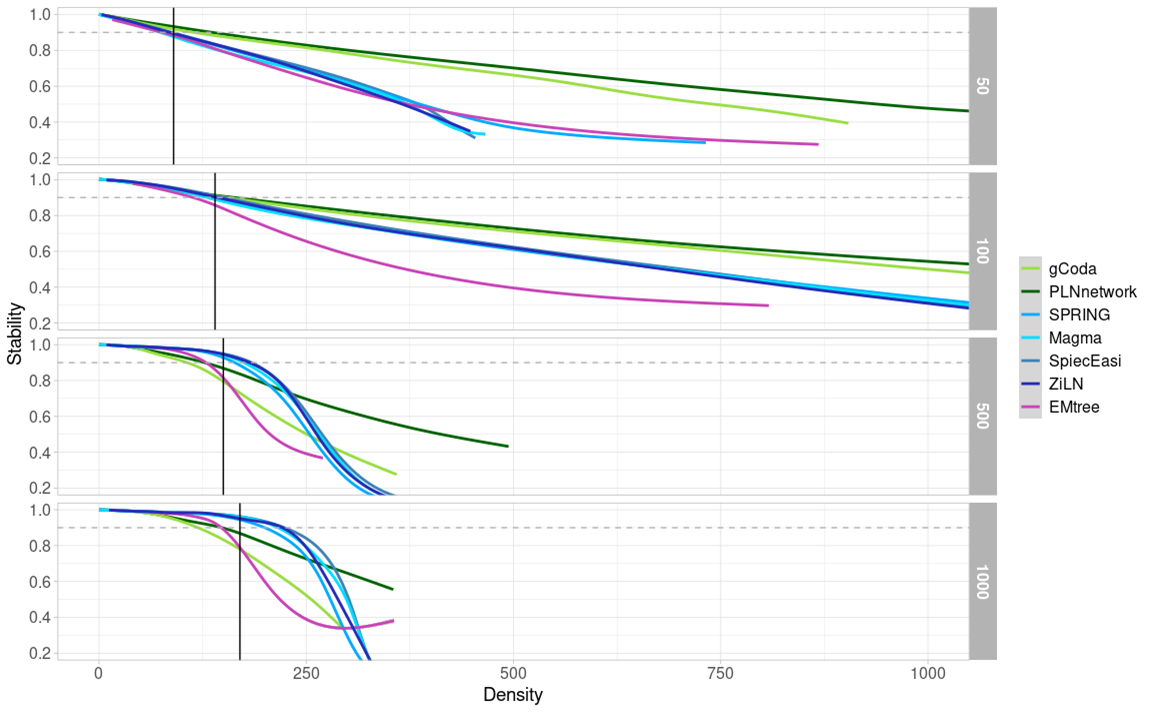

Supplement: S6 Fig — Each point in the curve corresponds to a different value of λ. The grey dashed horizontal line represents the target mean stability value (0.90) and the black vertical one, the associated density. (TIF) [file pcbi.1012627.s007.tif]

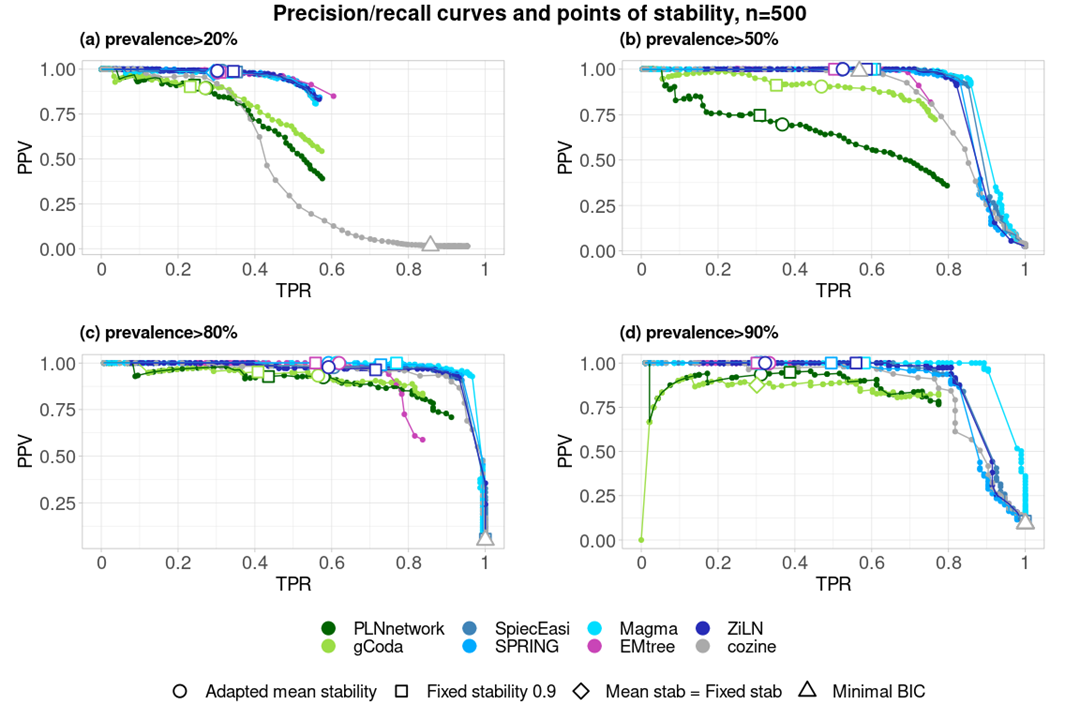

Supplement: S7 Fig — (a)0.20 (b)0.50 (c)0.8 (d)0.9 (see Fig 3 for details). (TIF) [file pcbi.1012627.s008.tif]

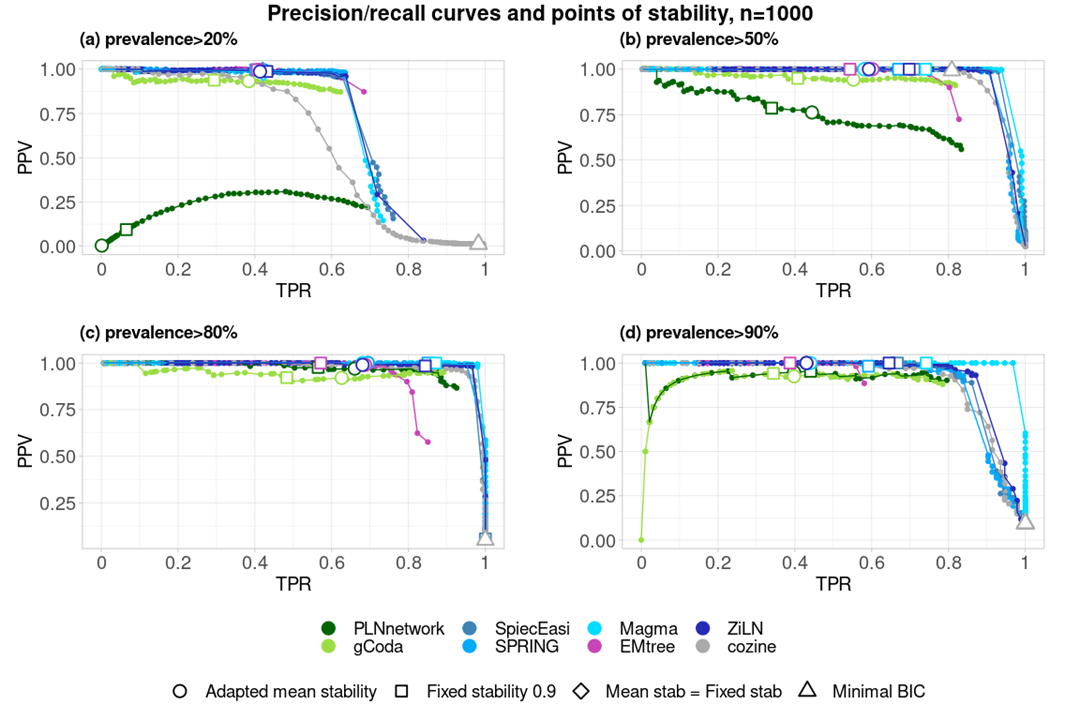

Supplement: S8 Fig — (a)0.20 (b)0.50 (c)0.8 (d)0.9 (see Fig 3 for details). (TIF) [file pcbi.1012627.s009.tif]

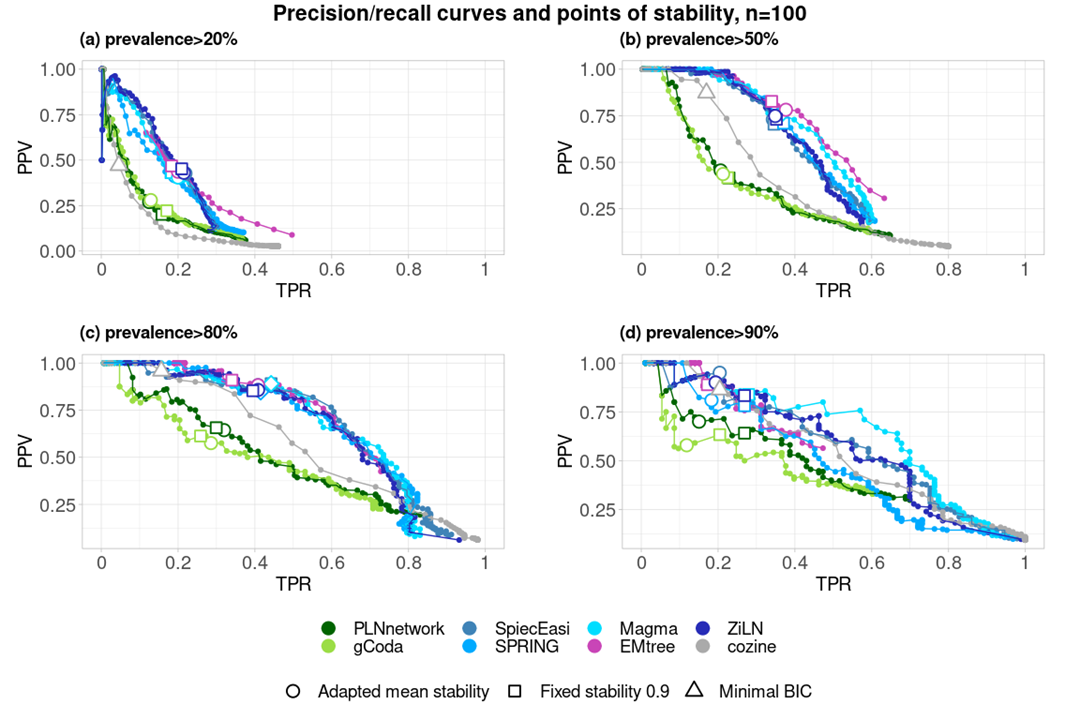

Supplement: S9 Fig — (a)0.20 (b)0.50 (c)0.8 (d)0.9 (see Fig 3 for details). (TIF) [file pcbi.1012627.s010.tif]

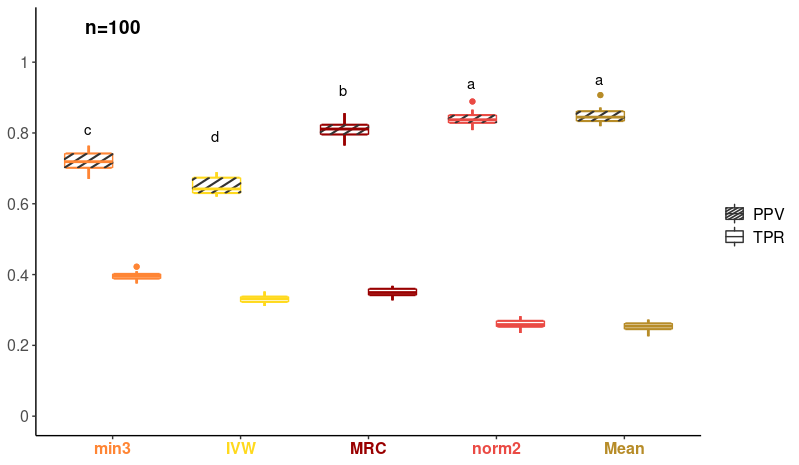

Supplement: S10 Fig — Striped (resp. no-strip) boxplots show PPV (resp. TPR) values. (TIF) [file pcbi.1012627.s011.tif]

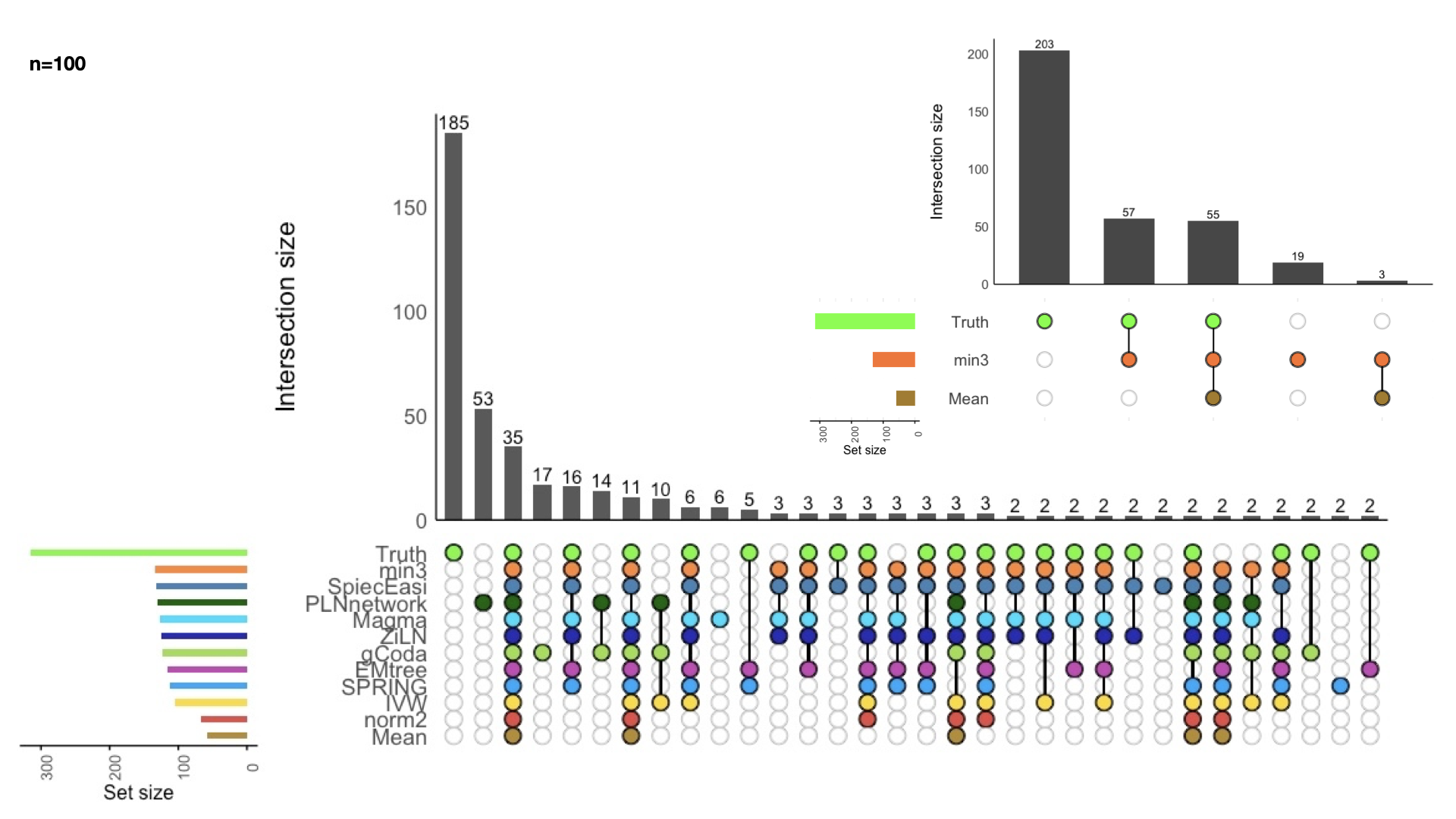

Supplement: S11 Fig — (TIF) [file pcbi.1012627.s012.tif]

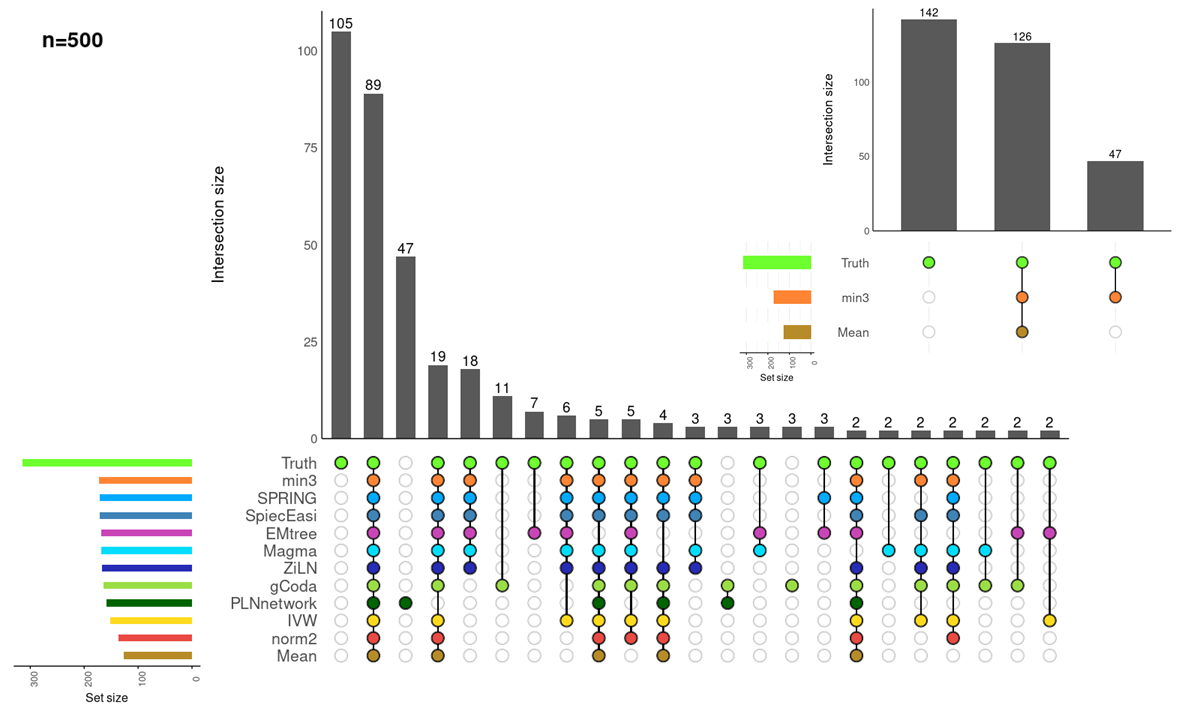

Supplement: S12 Fig — (TIF) [file pcbi.1012627.s013.tif]

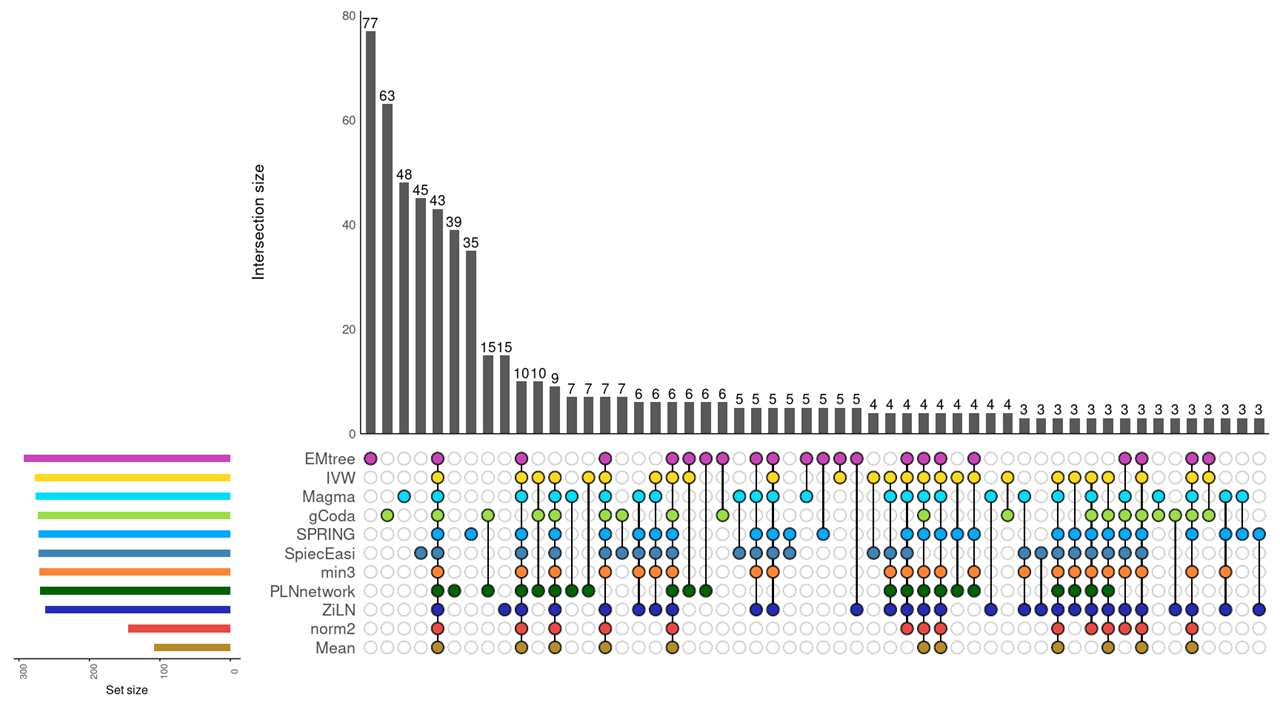

Supplement: S13 Fig — (TIF) [file pcbi.1012627.s014.tif]

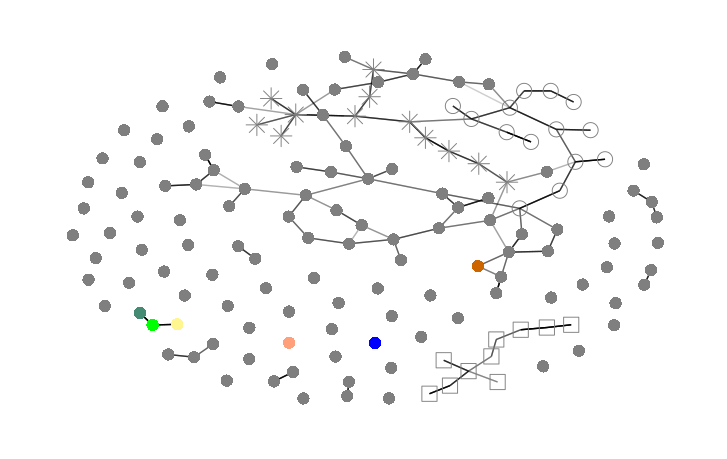

Supplement: S14 Fig — The guilds are represented by *, ∘ and □ and species from the cirrhotic guild are highlighted in color using the same color code as in Fig 6. (TIF) [file pcbi.1012627.s015.tif]
