# Supplementary material for: OneNet—One network to rule them all: Consensus network inference from microbiome data
Source: PLoS Comput Biol. 2024 Dec 6;20(12):e1012627. doi: 10.1371/journal.pcbi.1012627 (PMC11654977; doi:10.1371/journal.pcbi.1012627)
Supplement: S1 File — (PDF) [file pcbi.1012627.s016.pdf]

OneNet – One network to rule them all: consensus network inference from microbiome data

Camille Champion[1◑], Raphaëlle Momal[1◑], Emmanuelle Le Chatelier[1], Mathilde Sola[1], Mahendra Mariadassou [2], Magali Berland[1*]

1 Université Paris-Saclay, INRAE, MGP, 78350, Jouy-en-Josas, France
2 Université Paris-Saclay, INRAE, MaIAGE, 78350, Jouy-en-Josas, France

◑These authors contributed equally to this work.
* magali.berland@inrae.fr

Supplementary methods

We detail here each step of the whole network inference strategy. As microbiome abundance data don't directly fit within the gaussian framework, three main workarounds are commonly used: data transformation, models based on alternative distributions and models based on latent variables:

Transformations. A small constant is added to each abundance before log-transforming them. However, this transformation does not stabilize data variability because the log-transformed abundances scale with sequencing depths and covary with it, making dependencies modeling tricky. On the contrary, the Centered Log Ratio (CLR) transformation [3] guarantees the study of dependencies. It is however highly criticized when data contain a high proportion of zeros: proportions higher than 90% are typical in whole-metagenome or amplicon sequencing data. To circumvent this problem, [1] introduced a modified version of the CLR transformation (mCLR) that respects the original ordering of the data but doesn't account anymore for the compositional nature of the data, the primary motivation of the CLR transformation. An alternative to compositional transformations is to use a normalization factor, such as Geometric Mean of Pairwise Ratios (GMPR) [4], Relative Log Expression (RLE) [5] and others like Wrench normalization factors [6] or Cumulative Sum Scaling [7]. The GMPR normalization is designed for abundances with a high proportion of zero values. It compares pairs of samples based only on the species they share, and considers the geometric mean of the median ratio. This makes this technique robust to both differentially abundant species and extreme values. When two samples do not share any species, the computation of GMPR fails (this happens when samples come from very contrasted conditions with no or limited species overlap). In this case, Relative Log Expression (RLE) normalization method can be used. This method is based on the assumption that most of the species are not differentially abundant. However, this normalization factor fails when no single species is shared across all samples, which is frequently the case in microbiome data. A modified version of RLE only considers positive abundances to avoid this drawback.

Distributions and models. The second workaround is to use models adapted to abundance data characteristics : overdispersion (excess of variability in the data) and zero-inflation (excess of zeros). The **Poisson-log normal (PLN) model** [2] is designed for the analysis of abundance tables. It accounts for both structuring factors and potential interactions between the species. In the presence of overdispersion, the Poisson regression model is not adequate and can lead to biased parameter estimates

and unreliable standard errors estimates. The **Negative Binomial (NB) model** is then often used [8]. Both models can be seen as compound Poisson model (with a lognormal for the PLN distribution and Gamma for the NB) that are overdispersed compared to a *vanilla* Poisson distribution but the PLN is multivariate and can account for correlations between abundances. Contrary to the NB model, the **zero-inflated model** [9] is often motivated by an excess of zeros in the data, but less flexible than the zero outcome model. An intuitive approach to analyzing zero-inflated abundance data is to view the data as arising from a mixture distribution of a point mass distribution at zero and an abundance distribution. **Hurdle models** [10] are a class of models for abundance data that help handle excess zeros and overdispersion. In contrast to Zero inflated-models, hurdle models capture both an excess or a lack of zeros in the dataset. The **zero-inflated negative binomial (ZINB) model** [11], obtained by applying ZI to NB model, takes into account both overdispersion and excess of zeros. Finaly, **copulas** are a multivariate cumulative distribution functions for which the marginal probability distribution of each variable is uniform on the interval $[0, 1]$. As they fully describe the dependency structure, models with copulas allow to separate the modeling of marginal distributions (*e.g.* overdispersed, with excess zeros, etc) from the modeling of dependencies. Recent developments used gaussian copula coupled with arbitrary discrete marginal distributions to study multivariate abundance data [12]. [13] showed that Gaussian copulas are a relevant and promising approach to the problem of network inference from abundance data, even if the computational cost is higher than for other methods. One way of taking advantage of the copula theory without having to actually estimate the joint distribution is to use copulas as a sophisticated data transformation technique to transform abundances into pseudo-Gaussian data.

Latent variables. The third popular idea is to model multivariate discrete data using latent variables and push the depdendency back to the latent layer. Latent variables models have recently received increasing attention as they provide a convenient way to model the dependence structure between species. Two specifications of latent variables stand out in community ecology [14]: the **Multivariate Generalized Linear Mixed Model (GLMM)** [15, 18], and the **Latent Variable Model** (LVM) [16, 17]. The difference between these models lies in the dimension of their respective random effects: there are as many latent variables as there are species in the GLMM, whereas in the LVM their number is a parameter of the model.

Most methodologies to infer networks from abundance data first use a rationale (data transformation, latent variable modeling, etc.) to solve the problem of network inference in the Gaussian setting. There, they take advantage of the GGM framework to perform network inference using penalized likelihood or tree-based approaches to estimate the precision matrix, from which is finally derived the network.

Penalized likelihood approaches. There exist two main penalized approaches for the estimation of GGM: the graphical LASSO (glasso) [19], and the neighborhood selection, also called the Meinshausen-Bühlmann approach (MB) [20]. Both are penalized likelihood approaches which perform a sparse estimation of the precision matrix, either all at once for the glasso or row by row for MB.

Tree averaging approach. Another GGM inference method considered in this article is the tree averaging approach [21], which leverages specific algebraic properties to perform a complete and efficient exploration of the space of spanning tree structures. Note that this approach does not require the GGM Markov faithful property to hold. Each edge is given a posterior probability of being present in the network and those probabilities are thresholded to build the network.

References

1. Yoon G, Carroll RJ, Gaynanova I. Sparse Semiparametric Canonical Correlation Analysis for Data of Mixed Types. Biometrika. 2020;107(3):609–625.

2. Chiquet J, Mariadassou M, and Robin S, and al. The Poisson-Lognormal Model as a Versatile Framework for the Joint Analysis of Species Abundances. Frontiers in Ecology and Evolution. 2021;9.

3. Aitchison J. The Statistical Analysis of Compositional Data. Journal of the Royal Statistical Society: Series B (Methodological). 1982;44:139–160.

4. Chen L, Reeve J, Zhang L, Huang S, Wang X, Chen J, and al. GMPR: A Robust Normalization Method for Zero-Inflated Count Data with Application to Microbiome Sequencing Data. PeerJ. 2018;6.

5. Anders S, Huber W, and al. Differential expression analysis for sequence count data. Genome biology. 2010;11:10.

6. Senthil Kumar M, Slud EV, Okrah K, Hicks SC, Hannenhalli S, Corrada Bravo H, and al. Analysis and correction of compositional bias in sparse sequencing count data. BMC Genomics. 2018;19:799.

7. Paulson JN, Colin Stine O, Corrada Bravo H, Pop M, and al. Differential abundance analysis for microbial marker-gene surveys. Nat Methods. 2013;10:1200-1202.

8. Forbes C, Evans M, Hastings N, Peacock B, and al. Statistical distributions. John Wiley and Son. 2010.

9. Greene WH. Accounting for Excess Zeros and Sample Selection in Poisson and Negative Binomial Regression Models. Research Papers in Economics. 1994.

10. Cragg JG. Some Statistical Models for Limited Dependent Variables with Application to the Demand for Durable Goods. Econometrica. 1971;39:829-844.

11. Cheung YB. Zero-inflated models for regression analysis of count data: a study of growth and development. Statistics in Medicine. 2002;21.

12. Anderson MJ, de Valpine P, Punnett A, Miller AE, and al. A pathway for multivariate analysis of ecological communities using copulas. Ecology and evolution. 2019;9:3276–3294.

13. Popovic GC, Warton DI, Thomson FJ, Hui KFC, Moles AT, and al. Untangling direct species associations from indirect mediator species effects with graphical models. Methods in Ecology and Evolution. 2019;10:1571–1583.

14. Warton DI, Blanchet FG, O'Hara RB, Ovaskainen O, Taskinen S, Walker SC, Hui KFC, and al. So many variables: joint modeling in community ecology. Trends in Ecology and Evolution. 2015;30:766–779.

15. Ovaskainen O, HottolaJ, Siitonen J, and al. Modeling species co-occurrence by multivariate logistic regression generates new hypotheses on fungal interactions. Ecology. 2010;91:2514–2521.

16. Ovaskainen O, Abrego N, Halme P, Dunson D, and al. Using latent variable models to identify large networks of species-to-species associations at different spatial scales. Methods in Ecology and Evolution. 2016;7:549–555.

17. Ovaskainen O, Tikhonov G, Norberg A, Guillaume F, Duan L, Dunson D, Roslin T, Abrego N, and al. Using latent variable models to identify large networks of species-to-species associations at different spatial scales. Ecology Letters. 2017;20:561–576.

18. Pollock LJ, Tingley R, Morris WK, Golding N, O'Hara RB, Parris KM, VeskPA, McCarthy MA, and al. Understanding co-occurrence by modelling species simultaneously with a joint species distribution model (jsdm). Methods in Ecology and Evolution. 2014;5:397–406.

19. Friedman J, Hastie T, Tibshirani R, and al. Sparse inverse covariance estimation with the graphical lasso. Biostatistics. 2008;9:432–441.

20. Meinshausen N, Bühlmann P, and al. High-dimensional graphs and variable selection with the lasso. The annals of statistics. 2006;1436–1462.

21. Meila M, Jordan MI, and al. Learning with mixtures of trees. Journal of Machine Learning Research. 2000;1–48.
